# Development and Internal Validation of a Novel Prognostic Score in Metastatic Colorectal Cancer: A Comparative Retrospective Cohort Study with the Glasgow Prognostic Score and Gustave Roussy Immune Score

**DOI:** 10.3390/jcm15135074

**Published:** 2026-06-29

**Authors:** Simay Çokgezer, Senem Karabulut

**Affiliations:** Department of Medical Oncology, Institute of Oncology, Istanbul University, Millet Street, Çapa Campus, Fatih, 34093 Istanbul, Turkey; drsenemkarabulut@gmail.com

**Keywords:** Glasgow Prognostic Score, Gustave Roussy Immune Score, metastatic colorectal cancer, prognostic score

## Abstract

**Background**: Reliable prognostic stratification is essential in metastatic colorectal cancer (mCRC), yet current inflammation-based scores do not fully integrate systemic inflammation, tumor burden, and clinical characteristics. **Objective**: This study aimed to develop a novel prognostic score integrating clinical, tumor burden, and inflammatory parameters in patients with mCRC and to compare its performance with the Glasgow Prognostic Score (GPS) and Gustave Roussy Immune Score (GRIm). **Materials and Methods**: This retrospective single-center study included 310 patients with mCRC treated between 2015 and 2025. GPS and GRIm were calculated. The novel prognostic score was constructed using seven equally weighted prognostic variables encompassing clinical characteristics, tumor burden, and laboratory parameters. Survival analyses were performed using Kaplan–Meier and Cox regression. Model performance was assessed using AUC, reclassification, calibration, and decision curve analysis. **Results**: Most patients had ECOG PS 0–1 (96.1%), left-sided tumors (76.1%) and synchronous metastases (79.0%). The median follow-up was 23.6 months. Both GPS and GRIm were independent prognostic factors for OS. GPS demonstrated superior discriminative performance compared with GRIm (AUC for 24-month OS: 0.697 vs. 0.620; *p* = 0.005). The novel score stratified median OS into low-, intermediate-, and high-risk groups as 40.9, 25.4, and 11.5 months, respectively (*p* < 0.001). The novel model outperformed GPS and GRIm (AUC: 0.768) and improved reclassification. Bootstrap-based internal validation demonstrated low optimism and good calibration. **Conclusions**: The novel prognostic score integrating clinical, inflammatory, and tumor burden parameters demonstrated superior prognostic performance compared with GPS and GRIm in mCRC. This score may serve as tool for individualized risk stratification.

## 1. Introduction

Colorectal cancer (CRC) represents a major global health burden as the third most commonly diagnosed malignancy and the second leading cause of cancer-related mortality worldwide [1]. More than 1.9 million new cases are diagnosed annually, and approximately 20% of patients present with metastatic disease at the time of diagnosis [2]. In addition, nearly 50% of patients initially diagnosed with localized disease eventually develop distant metastases during the course of the disease. Despite advances in surgical techniques, modern chemotherapy combinations, and targeted therapies, the 5-year survival rate in patients with metastatic colorectal cancer (mCRC) remains below 15% [3]. The marked biological heterogeneity of metastatic disease and the substantial variability in treatment response have increased the need for easily applicable and cost-effective biomarkers capable of providing more accurate prognostic prediction [4]. In this context, the identification of reliable prognostic indicators that can be integrated into routine clinical practice carries significant clinical value for improving patient risk stratification and individualizing treatment strategies [5].

Cancer progression is determined not only by the proliferative capacity of tumor cells but also by the systemic inflammatory interaction between the host and tumor cells [6]. Systemic inflammation modulates the tumor microenvironment through inflammatory cell infiltration and cytokine release, thereby promoting invasion and metastatic processes [7]. Hypoalbuminemia and malnutrition, which frequently accompany this pathophysiological state, further impair host immune surveillance mechanisms and directly worsen survival outcomes [6,8]. The prognostic significance of inflammatory and nutritional parameters has led to the development of several prognostic scoring systems based on the integration of variables such as neutrophils, lymphocytes, and albumin [5,6,8,9,10]. These laboratory parameters, which are readily available in routine clinical practice, provide cost-effective, objective, and minimally invasive assessment tools [5,9,11]. Inflammation-based scoring systems therefore hold considerable clinical potential for optimizing risk stratification and individualizing treatment strategies in patients with metastatic disease [5,6,12].

Among the models integrating systemic inflammatory response and nutritional status, the Glasgow Prognostic Score (GPS) is one of the most extensively validated systems with established clinical significance [6,13]. Based on the combination of C-reactive protein (CRP) and serum albumin levels, this scoring system provides an objective measure of the host inflammatory burden across various solid tumor types, including CRC [13]. In the mCRC literature, particularly among patients receiving targeted therapies such as bevacizumab, elevated GPS values have been demonstrated to be an independent adverse prognostic factor for overall survival (OS) and an effective parameter for predicting clinical outcomes [6,8,13]. Owing to its simplicity, low cost, and high clinical applicability, this system offers a significant advantage in patient risk stratification, as it can be easily derived from routine laboratory tests [6]. However, because GPS primarily focuses on humoral inflammatory markers, it may be limited in fully reflecting the complex dynamics of the cellular immune response within the tumor microenvironment [13]. Furthermore, as GPS consists of only two parameters (CRP and albumin), it does not incorporate other critical clinical dimensions known to influence prognosis in mCRC, such as tumor burden, performance status, and the ability to receive treatment.

The limitations of GPS, particularly its focus on humoral inflammatory markers, have increased the clinical relevance of alternative models such as the Gustave Roussy Immune Score (GRIm), which provides a more comprehensive assessment of host–tumor interaction. Initially developed as an objective alternative to the Royal Marsden Hospital (RMH) score to optimize patient selection in phase I immunotherapy clinical trials, GRIm is based on the combination of serum albumin, neutrophil-to-lymphocyte ratio (NLR), and lactate dehydrogenase (LDH) levels [14]. The prognostic utility of this scoring system in predicting survival has been validated through large-scale meta-analyses across various solid tumor types, including lung, pancreatic, gastric, and esophageal cancers [11,14,15]. Current evidence in the mCRC literature indicates that a high GRIm score is directly associated with poorer OS and progression-free survival (PFS) outcomes and represents an independent adverse prognostic factor. Although its prognostic significance has been particularly emphasized in subgroups receiving anti-EGFR-based therapies, its predictive value in treatment regimens involving agents such as bevacizumab remains insufficiently clarified. Compared with GPS, the principal theoretical advantage of GRIm lies in its incorporation not only of systemic inflammation and nutritional status but also of NLR, reflecting cellular immunogenicity, and LDH levels, which serve as indicators of tumor metabolic activity and tumor burden [14]. Nevertheless, studies directly comparing the prognostic performance of these two scoring systems in the mCRC population remain limited. Moreover, similar to GPS, GRIm is also based exclusively on laboratory parameters and does not incorporate clinically relevant variables such as Eastern Cooperative Oncology Group Performance Status (ECOG PS), history of primary tumor surgery, anatomical distribution of metastatic disease, or tumor burden indicators such as carcinoembryonic antigen (CEA).

The primary aim of this study was to develop a novel prognostic score integrating clinical, pathological, and laboratory parameters in patients with mCRC and to evaluate its discriminative and calibration performance for OS using internal validation methods. As a secondary objective, we aimed to directly compare the independent prognostic value of GPS and GRIm scores, both representing combined indicators of systemic inflammation and nutritional status, for OS and PFS within the same patient population, and to compare the prognostic performance of the newly developed score against these established tools. Current literature provides limited data directly comparing GPS and GRIm within the same cohort, and there remains an unmet need for validated and practical prognostic tools integrating inflammatory biomarkers with clinical parameters in mCRC. In this regard, the findings of our study are expected to provide a readily calculable risk stratification tool that may support individualized treatment planning in routine clinical practice and contribute novel evidence to the field of inflammation-based prognostic assessment in mCRC.

## 2. Materials and Methods

### 2.1. Study Design and Patient Population

This retrospective single-center cohort study included patients diagnosed with mCRC who were followed and treated at the Department of Medical Oncology, Istanbul University Institute of Oncology, between January 2015 and December 2025. The study was conducted in accordance with the principles of the Declaration of Helsinki and was approved by the Ethics Committee of Istanbul University Istanbul Faculty of Medicine. Due to the retrospective study design, the requirement for informed consent was waived by the ethics committee.

The inclusion criteria were as follows: (1) histopathologically confirmed diagnosis of colorectal adenocarcinoma, (2) presence of radiologically or pathologically confirmed metastatic disease either at diagnosis or during follow-up, (3) availability of complete blood count, serum albumin, CRP, LDH, and CEA data enabling calculation of GPS, GRIm, and the novel prognostic score prior to first-line systemic therapy, (4) accessibility of regular clinical follow-up data, and (5) sufficient data for treatment response and survival analyses. The exclusion criteria were as follows: (1) presence of active infection, chronic inflammatory disease, or hematological disorder, (2) concurrent second primary malignancy, (3) missing laboratory or follow-up data, (4) use of immunosuppressive therapy or systemic corticosteroid treatment, and (5) patients who received only best supportive care.

**Figure 1:**
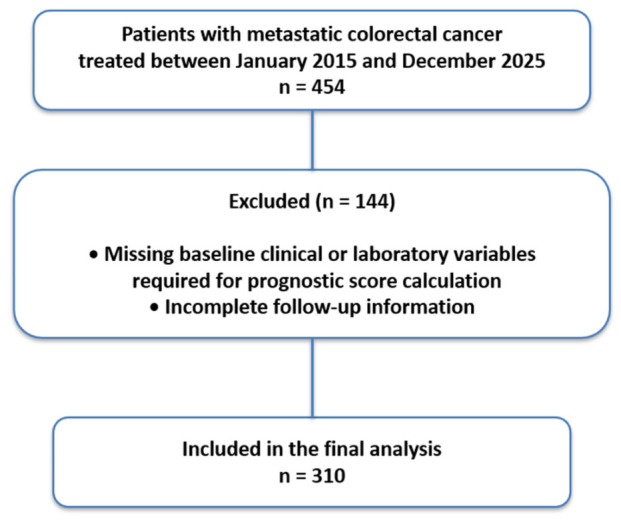
Flow diagram of patient selection.

A total of 454 consecutive patients with mCRC were screened during the study period. After applying the predefined eligibility and exclusion criteria, 310 patients were included in the final analysis. The patient selection process is summarized in Figure 1.

### 2.2. Data Collection and Clinical Variables

Patient data were retrospectively collected using the electronic medical record system, chemotherapy unit records, and archived patient files. Demographic data included age, sex, performance status (ECOG PS), comorbidities, and date of diagnosis. Tumor-related variables included primary tumor localization (right colon, left colon, rectum), histopathological subtype, tumor grade, RAS/BRAF mutation status, microsatellite instability (MSI/MMR) status, metastatic sites, metastatic burden, and previous surgical interventions. Treatment-related variables included administered chemotherapy regimens, use of biological agents and immunotherapy, treatment lines, treatment response, and progression dates.

Laboratory parameters were obtained from measurements performed within the closest 14 days prior to the initiation of systemic therapy. CEA levels were recorded from the nearest measurement obtained either at the time of metastatic disease diagnosis or within 28 days before initiation of first-line treatment. ECOG PS was determined based on the oncologist’s assessment at the initiation of first-line systemic therapy.

### 2.3. GPS Assessment

GPS was calculated based on serum CRP and albumin levels. Patients with CRP > 10 mg/L and albumin < 3.5 g/dL were classified as GPS 2; patients with only one abnormal parameter were classified as GPS 1; and patients with both parameters within normal ranges were classified as GPS 0. This score was used as a combined indicator of systemic inflammation and nutritional status.

### 2.4. GRIm Assessment

The GRIm score was calculated based on serum albumin, NLR, and LDH levels. One point was assigned for each of the following criteria: albumin < 3.5 g/dL, NLR > 6, and LDH level above the upper limit of normal. The total score ranged from 0 to 3. Due to the limited number of patients in the higher score categories, in order to preserve statistical power and maintain consistency with the literature, scores were categorized into low (0–1) and high (2–3) GRIm groups.

### 2.5. Development of the Novel Prognostic Score

The novel prognostic score was designed as the sum of binary risk factors integrating clinical, pathological, and laboratory parameters. The candidate variable pool consisted of 17 parameters previously demonstrated to be associated with OS in the mCRC literature: age ≥ 65 years, ECOG PS ≥ 2, presence of comorbidity, right-sided colon localization, RAS mutation status, synchronous metastasis, ≥2 metastatic sites, liver metastasis, lung metastasis, peritoneal metastasis, absence of primary tumor surgery, albumin < 3.5 g/dL, CRP > 10 mg/L, NLR > 3, LDH above the upper limit of normal, lymphocyte count <1.0 × 10^9^/L, and CEA ≥ 5 ng/mL. Continuous variables were dichotomized using clinically established cutoff values adopted from previously validated prognostic models and the published CRC literature to maximize clinical applicability and facilitate bedside implementation of the proposed scoring system.

Variable selection was performed in two stages: (1) variables associated with survival in univariable Cox regression analysis were identified for preliminary selection; (2) these variables were subsequently included in a multivariable Cox model, and variables with *p* ≥ 0.05 were sequentially removed using the backward elimination method. To minimize the risk of overfitting, candidate variables were predefined based on clinical relevance. Nevertheless, because variable selection was performed within the study dataset, bootstrap-based internal validation was conducted to assess model stability.

Seven variables remained in the final model: ECOG PS ≥ 2, albumin < 3.5 g/dL, peritoneal metastasis, ≥2 metastatic sites, CEA ≥ 5 ng/mL, absence of primary tumor surgery, and CRP > 10 mg/L. These seven parameters were equally weighted (1 point for each factor) to generate the total score (0–7 points). This simplified scoring structure was adopted to facilitate routine clinical use despite modest differences in the estimated effect sizes of the retained predictors. Risk groups were categorized according to predefined cutoff values as low risk (0–1 points), intermediate risk (2–3 points), and high risk (≥4 points).

To evaluate the adequacy of the simplified equal-weighted scoring system, a β-coefficient-weighted alternative score was also constructed using the same final multivariable Cox model. Points were assigned proportionally to the regression coefficients by dividing each β-coefficient by the smallest retained coefficient and rounding to the nearest integer. The discriminative performance of the two scoring approaches was subsequently compared.

### 2.6. Endpoints

The primary endpoint was defined as OS and was calculated as the time from the date of metastatic disease diagnosis to death from any cause; patients who were alive at the end of follow-up were censored at the date of last follow-up. The secondary endpoint was PFS, defined as the time from the date of metastatic disease diagnosis to radiological or clinical progression or death from any cause; patients who were alive without progression were censored at the date of last follow-up. Treatment responses were assessed according to RECIST 1.1 criteria.

### 2.7. Statistical Analysis

Statistical analyses were performed using IBM SPSS Statistics (version 28.0; IBM Corp., Armonk, NY, USA) and R software (version 4.3.0). Continuous variables were presented as median (IQR) or mean ± standard deviation (SD) according to data distribution, while categorical variables were expressed as number (%). Comparisons between groups were performed using the chi-square or Fisher’s exact test for categorical variables and Student’s *t*-test or the Mann–Whitney U test for continuous variables.

Survival analyses were conducted using the Kaplan–Meier method, and groups were compared with the log-rank test. The effects of GPS, GRIm, and clinical variables on OS and PFS were evaluated using univariable and multivariable Cox regression analyses, and the results were reported as hazard ratios (HRs) with 95% confidence intervals (CIs). Due to multicollinearity, GPS and GRIm were not included in the same model and were instead evaluated in separate multivariable models. The proportional hazards assumption for the Cox regression models was assessed using Schoenfeld residual-based tests. The prognostic performance of the scores was assessed using Harrell’s C-index, time-dependent ROC analysis with AUC values, and comparisons using the DeLong test; 95% CIs for AUC values were calculated using 1000 bootstrap resamples.

The novel prognostic score was constructed as the equally weighted sum of variables selected from 17 binary candidate variables previously associated with survival in the mCRC literature, using univariable Cox analysis (*p* < 0.10) followed by multivariable Cox regression with backward elimination (sequential removal of variables with *p* ≥ 0.05). Patients were subsequently categorized into low-, intermediate-, and high-risk groups. The reclassification performance of the novel score compared with GPS and GRIm was evaluated using continuous net reclassification improvement (NRI) and integrated discrimination improvement (IDI). Model calibration was assessed using calibration curves generated with 1000 bootstrap resamples, together with calibration slope and intercept values. Clinical utility was evaluated using decision curve analysis. As no independent external validation cohort was available, only internal validation was performed. A nomogram based on the multivariable Cox regression model was developed for individualized survival prediction.

Subgroup analyses were performed according to biological agent type, RAS status, and primary tumor localization. A two-sided *p*-value < 0.05 was considered statistically significant for all analyses.

During the planning and execution of the statistical analyses, the authors used Claude (Anthropic, version 4.7; Anthropic PBC, San Francisco, CA, USA) as an AI-assisted tool to support the selection of appropriate statistical methods, generation and review of R code, and verification of analytical workflows. All analyses were ultimately performed in IBM SPSS Statistics and R as described above, and all AI-generated outputs, code, and interpretations were independently reviewed, verified, and validated by the authors. The authors take full responsibility for the integrity, accuracy, and interpretation of all statistical analyses presented in this study.

## 3. Results

### 3.1. Patient Characteristics

A total of 310 patients with mCRC who met the study criteria were included in the analysis. The median follow-up duration among surviving patients was 23.6 months (IQR 14.3–32.5; maximum 110 months). During follow-up, death occurred in 244 patients (78.7%) and disease progression was observed in 278 patients (89.7%). For the entire cohort, median OS was 20.0 months (IQR 10.6–30.9), while median PFS was 10.8 months (IQR 6.3–17.7). Baseline demographic, clinicopathological, and laboratory characteristics of the patients according to GPS and GRIm risk groups are presented in Table 1.

The majority of patients had good performance status (ECOG PS 0–1, 96.1%) and left-sided primary tumors (76.1%). Synchronous metastatic disease was present in 79.0% of patients, and the liver was the most common metastatic site (71.3%). RAS mutation was detected in 41.6% of patients. In first-line treatment, 62.3% of patients received anti-VEGF-based regimens, whereas 27.4% received anti-EGFR-based combination therapies.

Inflammatory and nutritional parameters differed significantly between GPS and GRIm risk groups. In the high GRIm group, median NLR (6.7 vs. 2.8), albumin (3.2 vs. 4.1 g/dL), CRP (56.4 vs. 9.2 mg/L), and LDH (324.5 vs. 207.5 U/L) levels were significantly different compared with the low GRIm group (all *p* < 0.001). A similar trend was observed across GPS categories as a distinct three-tier gradient (for GPS 0 vs. 1 vs. 2: CRP 3.4 vs. 22.8 vs. 61.0 mg/L; albumin 4.3 vs. 4.0 vs. 3.2 g/dL; all *p* < 0.001).

Indicators of tumor burden and clinical severity increased in parallel with both scoring systems. In the high GRIm group, the proportion of patients with ≥2 metastatic sites was significantly higher (62.5% vs. 43.1%; *p* = 0.018), and median CEA levels were elevated (48.0 vs. 19.0 ng/mL; *p* = 0.022). Across GPS groups, the rates of synchronous metastatic disease (68.6% vs. 85.7% vs. 91.5%; *p* < 0.001), ≥2 metastatic sites (35.8% vs. 53.2% vs. 57.4%; *p* = 0.004), and absence of adjuvant chemotherapy (65.7% vs. 82.5% vs. 87.2%; *p* < 0.001) increased significantly with increasing score. The proportion of patients who had not undergone primary tumor surgery also differed significantly between groups (36.5% vs. 61.9% vs. 53.2%; *p* < 0.001). In addition, ECOG PS ≥ 2 was more frequent in the high GPS group (10.6% vs. 2.2%; *p* = 0.028), and median age was significantly higher (65 vs. 59 years; *p* = 0.042).

### 3.2. Impact of GPS and GRIm Scores on Survival

Kaplan–Meier analysis demonstrated that both scores significantly stratified OS (Table 2, Figure 1). Median OS was 23.7 months in the low GRIm group and 12.5 months in the high GRIm group (log-rank *p* < 0.001). Across GPS groups, a clear three-tier gradient was observed, with median OS values of 29.7, 20.4, and 10.7 months, respectively (log-rank *p* < 0.001), while 24-month OS rates declined from 65.2% to 17.0%.

**Figure 2:**
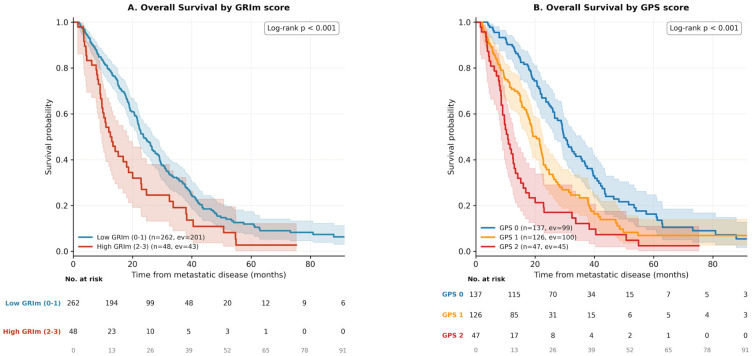
Overall survival according to GRIm (**A**) and GPS groups (**B**).

Distinct prognostic patterns were observed between GPS and GRIm scores in PFS analyses. GPS significantly stratified PFS outcomes (median PFS: 13.8 vs. 10.2 vs. 8.6 months; log-rank *p* < 0.001), whereas no statistically significant difference in PFS was observed between GRIm groups (11.7 vs. 9.2 months; log-rank *p* = 0.108) (Figure 2).

In multivariable Cox models adjusted for clinical covariates, both scores retained independent prognostic significance for OS (Table 3). Compared with the low GRIm group, the high GRIm group was associated with a 79% increased risk of mortality (adjusted HR [aHR] 1.79, 95% CI 1.22–2.64; *p* = 0.003). For GPS, a graded dose–response relationship was observed, with GPS 1 associated with an aHR of 1.52 (*p* = 0.006) and GPS 2 with an aHR of 2.88 (*p* < 0.001). In PFS analyses, GPS maintained independent prognostic significance (GPS 2 vs. 0: aHR 1.62; *p* = 0.024), whereas GRIm lost statistical significance (aHR 1.21; *p* = 0.308). ECOG PS ≥ 2, ≥2 metastatic sites, and absence of primary tumor surgery emerged as strong independent prognostic factors for OS in both models.

### 3.3. Comparison of the Discriminative Performance of GPS and GRIm

**Figure 3:**
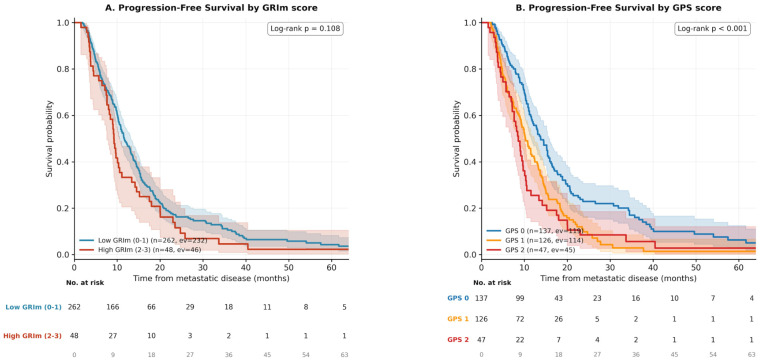
Progression-free survival according to GRIm (**A**) and GPS groups (**B**).

In the time-dependent ROC analysis for 24-month OS, GPS demonstrated significantly superior discriminative performance compared with GRIm (AUC 0.697 vs. 0.620; DeLong *p* = 0.005) (Figure 3). Univariable Harrell’s C-index analysis yielded consistent findings, with a C-index of 0.635 for GPS and 0.597 for GRIm (Supplementary Table S1). For PFS endpoints, the AUC values of both scores remained within the range of 0.60–0.61, and their discriminative performances were not statistically distinguishable from each other (DeLong *p* > 0.36). In multivariable models, the model incorporating GPS also demonstrated higher discriminative performance than the model including GRIm (C-index for OS: 0.695 vs. 0.675).

### 3.4. Subgroup Analyses

Subgroup analyses according to first-line biological agent type, RAS status, and primary tumor localization demonstrated that the prognostic effects of both scores varied significantly across subgroups (Table 4). Among patients receiving anti-EGFR therapy, both GRIm and GPS showed particularly strong prognostic effects (high GRIm: HR 6.58, *p* < 0.001; GPS 2: HR 6.58, *p* < 0.001). In patients treated with anti-VEGF-based regimens, GPS retained prognostic significance (HR 1.97, *p* < 0.001), whereas no statistically significant association was observed for GRIm (HR 1.45, *p* = 0.076). In the RAS wild-type subgroup, both scores remained significant, whereas in the mutant subgroup the prognostic effect of GRIm did not reach statistical significance (*p* = 0.052). Both scores provided strong discrimination in patients with left-sided tumors, while no significant results were observed among the limited number of patients with right-sided tumors (n = 72).

### 3.5. Derivation of the Novel Prognostic Score

Among the 17 candidate variables included in the initial variable pool, 11 were significantly associated with OS at the *p* < 0.10 level in univariable Cox analysis. First-line biological therapy was also evaluated among the candidate predictors, but neither anti-VEGF nor anti-EGFR therapy met the predefined criterion for inclusion in the multivariable model. Following backward elimination, seven independent prognostic factors remained in the final model: ECOG PS ≥ 2 (aHR 2.41), albumin < 3.5 g/dL (aHR 1.91), peritoneal metastasis (aHR 1.54), ≥2 metastatic sites (aHR 1.53), CEA ≥ 5 ng/mL (aHR 1.49), absence of primary tumor surgery (aHR 1.45), and CRP > 10 mg/L (aHR 1.45) (all *p* < 0.05) (Table 5). Each factor was assigned 1 point, resulting in a total score ranging from 0 to 7.

### 3.6. Performance and Validation of the Novel Score

**Figure 4:**
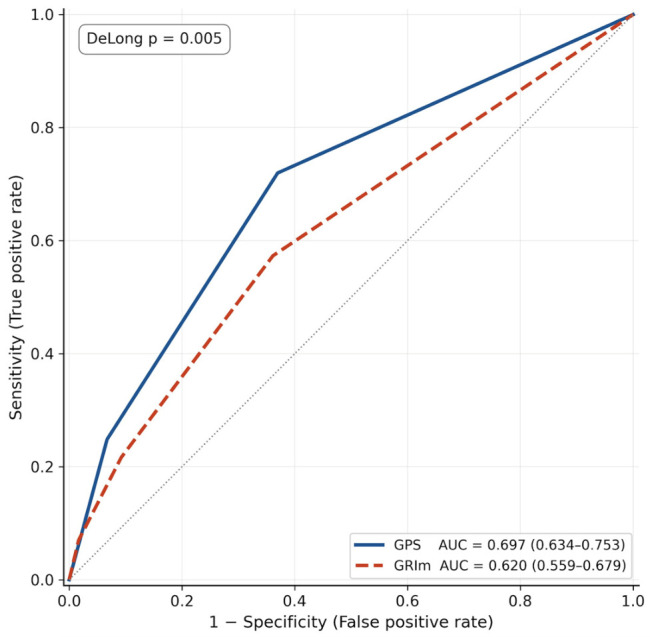
Time-dependent ROC curves of GPS and GRIm for predicting 24-month overall survival. (The black dashed line represents the line of no discrimination (AUC = 0.5)).

Distinct survival gradients were observed across risk groups (Table 6, Figure 4). In the low-risk group (0–1 points, n = 67), median OS was 40.9 months with a 24-month OS rate of 72.8%; in the intermediate-risk group (2–3 points, n = 155), median OS was 25.4 months with a 24-month OS rate of 52.8%; and in the high-risk group (≥4 points, n = 88), median OS was 11.5 months with a 24-month OS rate of 13.6% (log-rank *p* < 0.001). The nearly fourfold survival difference between the low- and high-risk groups supports the clinically meaningful stratification provided by the score.

**Figure 5:**
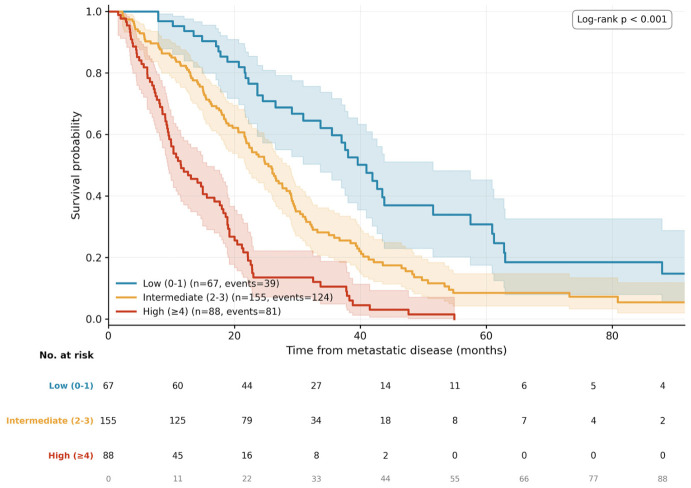
Overall survival according to the novel prognostic score risk groups.

For 24-month OS, the novel score achieved an AUC of 0.768 (95% CI 0.710–0.820), demonstrating significantly superior discriminative performance compared with both GPS (AUC 0.697; DeLong *p* = 0.005) and GRIm (AUC 0.620; DeLong *p* < 0.001) (Figure 5). In reclassification analyses, the novel score provided improvement over GPS with a continuous net reclassification improvement (NRI) of +0.331 (95% CI +0.098 to +0.559) and integrated discrimination improvement (IDI) of +0.072 (95% CI +0.022 to +0.126), and over GRIm with an NRI of +0.416 (95% CI +0.183 to +0.642) and IDI of +0.148 (95% CI +0.081 to +0.220) (Supplementary Table S2).

**Figure 6:**
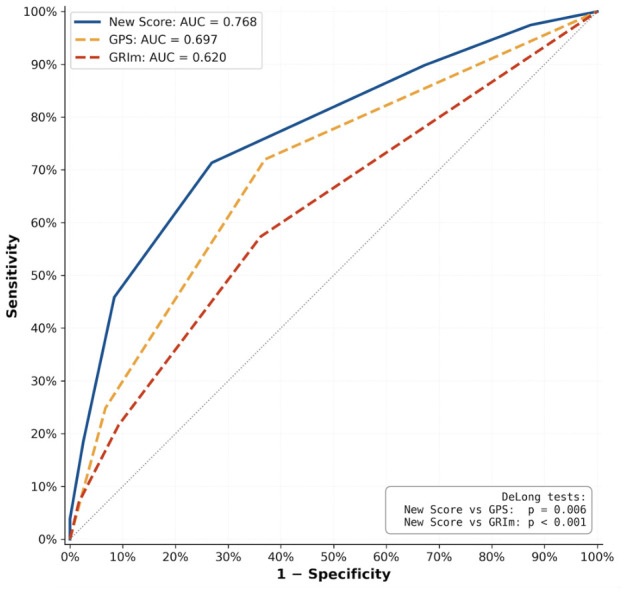
ROC comparison of the novel prognostic score, GPS, and GRIm for 24-month overall survival. (The dashed diagonal line represents the line of no discrimination (AUC = 0.5)).

**Figure 7:**
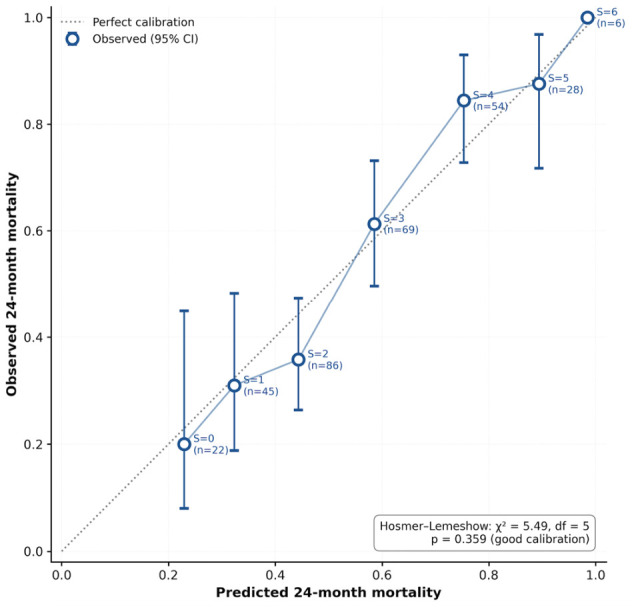
Bootstrap-based calibration plot of the novel prognostic score for 24-month overall survival.

Model calibration was favorable, with predicted 24-month mortality at each score level showing close agreement with Kaplan–Meier–observed mortality (Figure 6). The apparent calibration slope was 1.001 and the intercept was −0.027, indicating near-perfect agreement with the ideal calibration point (slope = 1, intercept = 0). After internal validation with 1000 bootstrap resamples, the optimism-corrected slope remained 0.909 and the intercept −0.029, suggesting a low degree of optimism in the model. In Steyerberg bootstrap internal validation, the apparent C-index was 0.681, the optimism estimate was close to 0.000, and the optimism-corrected C-index remained 0.681 (Supplementary Table S3). In decision curve analysis, the novel score provided substantially greater net clinical benefit than GPS and GRIm across the threshold probability range of 30–80% (Figure 7). A nomogram based on the β-coefficients of the final Cox model was constructed for individualized prediction of 24- and 36-month OS and was presented as a complementary analysis (Supplementary Figure S2).

**Figure 8:**
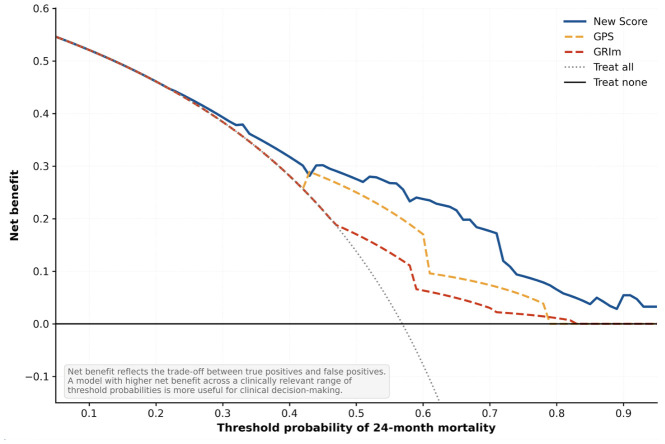
Decision curve analysis comparing the novel prognostic score with GPS and GRIm.

To assess the adequacy of the simplified equal-weighted scoring system, decision curve analysis was performed (Figure 8). In addition, a β-coefficient-weighted alternative score derived from the same final Cox model was evaluated for comparison. The two scoring approaches demonstrated comparable discriminative performance (24-month AUC: 0.768 vs. 0.767; DeLong *p* = 0.80). The apparent Harrell’s C-index was 0.681 for the equal-weighted model and 0.685 for the β-weighted model. However, bootstrap internal validation showed no optimism for the equal-weighted score, whereas the β-weighted model demonstrated mild optimism (+0.010), resulting in optimism-corrected C-indices of 0.681 and 0.675, respectively. The results of this comparison are summarized in Supplementary Table S4.

## 4. Discussion

In this retrospective cohort study, a novel prognostic score integrating clinical characteristics, tumor burden, and systemic inflammatory parameters was developed in patients with mCRC, and its performance was preliminarily evaluated using bootstrap-based internal validation methods. The developed score provided strong prognostic stratification for OS by clearly discriminating patients into low-, intermediate-, and high-risk groups; median OS was 40.9 months in the low-risk group, whereas it decreased to 11.5 months in the high-risk group. Furthermore, the novel model demonstrated superior discriminative performance compared with the established GPS and GRIm scores, which are based solely on inflammation-related parameters, achieving a higher AUC value for 24-month OS prediction together with significant improvement in reclassification performance. These findings suggest that prognosis in mCRC may be predicted more accurately through the combined assessment of not only systemic inflammation but also clinical determinants such as performance status, metastatic dissemination, and tumor burden.

The close relationship between systemic inflammation and tumor progression constitutes the biological basis of inflammation-based prognostic scores [14]. Elevated CRP levels induced by proinflammatory cytokines reflect aggressive tumor biology associated with tumor invasion, angiogenesis, and immune escape, whereas low serum albumin levels are considered indicators of both chronic inflammation and impaired nutritional status [6,13,14]. In our study, the inclusion of CRP elevation and hypoalbuminemia among the independent predictors in the novel prognostic model further supports the central role of inflammation and nutritional impairment in the prognosis of mCRC. Moreover, the progressive deterioration in CRP and albumin levels together with declining survival outcomes across increasing GPS categories demonstrates that these biomarkers provide clinically meaningful prognostic information.

In our study, GPS demonstrated superior discriminative performance compared with GRIm in predicting both OS and PFS. Despite the inclusion of parameters reflecting cellular immunity and tumor burden, such as NLR and LDH, within the GRIm score, the CRP- and albumin-based GPS exhibited more stable prognostic performance in the mCRC population. This finding suggests that systemic humoral inflammation and nutritional impairment may represent more dominant biological processes in determining prognosis in mCRC compared with cellular inflammatory ratios. In particular, the ability of CRP and albumin levels to more directly reflect chronic inflammation, hepatic reserve, and cancer-related catabolic burden may explain the more stable prognostic performance of GPS [6,8,13]. Furthermore, the limited number of studies directly comparing GPS and GRIm in mCRC cohorts indicates that our findings address an important gap in the literature and suggest that GPS may provide more reliable risk stratification in clinical practice. Because the primary objective of the present study was to compare the prognostic performance of GPS and GRIm and to develop an integrated score based on these established inflammation-based models, other published prognostic tools were not included in the comparative analyses. Future studies should evaluate the proposed score against a broader range of established prognostic models to further define its relative clinical value.

The prognostic relevance of systemic inflammation also extends to later lines of therapy. In patients with refractory mCRC receiving regorafenib, the Cancer-Inflammation Prognostic Index (CIPI), which combines CEA and NLR, has been identified as an independent prognostic marker for survival. These findings further support the concept that integrating inflammatory and tumor-related biomarkers improves prognostic stratification in mCRC [16]. Consistent with this evidence, our novel score incorporates inflammatory biomarkers together with clinical characteristics and tumor burden, providing a more comprehensive assessment of patient prognosis.

Beyond inflammation-based prognostic models, multidimensional prognostic scores have also been developed for specific mCRC subgroups. For example, in patients with metachronous colorectal peritoneal metastases undergoing cytoreductive surgery and hyperthermic intraperitoneal chemotherapy (CRS-HIPEC), a prognostic score integrating preoperative clinicopathological factors successfully stratified postoperative survival outcomes. Although this model was specifically designed for a highly selected surgical population, it highlights the growing need for individualized prognostic tools tailored to different clinical settings [17]. In contrast, our score was developed in an unselected real-world mCRC cohort, supporting its potential applicability across a broader patient population. More recently, lymph node-related indices have also been incorporated into prognostic nomograms for CRC patients with synchronous lung metastases. These models suggest that nodal parameters may provide additional prognostic information even in metastatic organ-specific subgroups [18]. Although our score did not incorporate lymph node-derived indices, the inclusion of metastatic burden and clinical status similarly reflects the importance of integrating multiple prognostic domains for comprehensive risk assessment in mCRC.

Artificial intelligence (AI)-based prognostic models have recently emerged as promising tools for personalized risk assessment in colorectal cancer. A recent systematic review highlighted the growing role of machine learning and deep learning algorithms in improving prognostic prediction and treatment decision-making across different stages of CRC [19]. Building upon these advances, an AI-augmented deep learning model was subsequently developed and externally validated in patients with mCRC receiving first-line therapy, successfully stratifying patients into distinct progression-free survival risk groups using routinely available clinical and laboratory variables [20]. Although our model was developed using conventional statistical methods, these findings suggest that future prognostic research may benefit from integrating AI-based approaches with established clinical and inflammation-based models to further improve individualized risk assessment.

Emerging biomarkers may further refine prognostic assessment in colorectal cancer. For example, recent evidence suggests that decreased butyrylcholinesterase levels are associated with an increased risk of postoperative complications following colorectal surgery, highlighting the potential value of biomarkers reflecting systemic inflammation and nutritional status [21]. Although butyrylcholinesterase was not evaluated in our study, future studies should investigate whether integrating such emerging biomarkers with established clinical and inflammatory parameters can further improve prognostic stratification in mCRC.

Subgroup analyses demonstrated that the prognostic impact of inflammation-based scoring systems may vary according to treatment regimen and molecular subtype. In particular, both GPS and GRIm showed strong prognostic significance among patients receiving anti-EGFR therapy, suggesting that host inflammatory status may influence not only tumor biology but also treatment response. The therapeutic efficacy of anti-EGFR monoclonal antibodies may involve not only receptor blockade but also immune activation through antibody-dependent cellular cytotoxicity mechanisms, which may explain the strong association between inflammatory burden and survival outcomes [22]. In contrast, although GPS retained prognostic significance in the anti-VEGF treatment subgroup, GRIm lost statistical significance, suggesting a complex interaction between angiogenesis and systemic inflammation. While CRP and albumin may provide a more stable reflection of chronic inflammation, hepatic reserve, and protein balance, thereby explaining the superiority of GPS in this subgroup, the GRIm components LDH and NLR may exhibit greater variability under bevacizumab treatment, potentially reducing discriminative performance [6,11,13,14]. Similarly, the weaker performance of GRIm in patients with RAS-mutant and right-sided tumors may be related to the intrinsic biological heterogeneity of these subgroups. Right-sided colon tumors are characterized by higher baseline inflammation, mucinous histology, and increased frequencies of RAS/BRAF mutations, all of which may reduce the discriminative capacity of inflammation-based scoring systems [1,14]. Nevertheless, the limited sample size within the right-sided tumor subgroup should also be considered when interpreting these findings. Collectively, these results support the importance of considering treatment modality and molecular subtype in the clinical application of inflammation-based prognostic tools.

The principal limitation of GPS and GRIm is that they largely restrict prognostic assessment to laboratory parameters and insufficiently reflect the overall clinical burden of the patient [14]. In contrast, the superior performance of the novel score developed in our study may derive from its integration of CRP and albumin levels representing systemic inflammation and nutritional status; ≥2 metastatic sites, peritoneal metastasis, and CEA levels reflecting tumor burden and anatomical dissemination; and ECOG PS together with history of primary tumor surgery reflecting the patient’s overall condition and treatment eligibility. Indeed, the independent prognostic significance of all these parameters in multivariable analysis supports the concept that prognosis in mCRC is determined not solely by inflammation, but by the combined effects of tumor biology, metastatic extent, and host reserve. In particular, peritoneal metastasis and high tumor burden likely reflect aggressive disease biology, whereas ECOG PS may represent treatment tolerance and overall physiological reserve, thereby enabling the model to capture clinical reality more comprehensively. As a consequence of this integrative approach, the novel score more effectively represented the marked biological heterogeneity of mCRC than systems based solely on inflammatory biomarkers and demonstrated superior discriminative performance compared with GPS and GRIm. Although ECOG PS ≥ 2 was retained as an independent predictor in the final model, the relatively small number of patients with ECOG PS ≥ 2 may have limited the precision of its estimated effect. Therefore, the prognostic contribution of ECOG PS should be confirmed in larger external cohorts.

One of the most notable features of the developed prognostic score is its ease of application in routine clinical practice despite its strong discriminative performance. The equally weighted structure of the model, with each prognostic factor assigned only 1 point, enables rapid risk stratification in daily oncology practice without the need for complex mathematical calculations. This approach may facilitate the standardization of prognostic assessment, particularly in busy clinical settings. Furthermore, the finding that the novel score provided greater net clinical benefit than GPS and GRIm across the 30–80% threshold probability range in decision curve analysis indicates that the model offers not only statistical accuracy but also meaningful clinical utility in decision-making. This may help guide clinicians in avoiding unnecessarily aggressive treatments associated with excessive toxicity in low-risk patients, while supporting the selection of more intensive treatment and closer follow-up strategies for high-risk patients.

Several limitations of this study should be acknowledged. First, the retrospective single-center design cannot completely eliminate the potential risk of bias related to patient selection and treatment heterogeneity. Nevertheless, the relatively large cohort of 310 patients managed over a 10-year period supports the statistical robustness of the findings. The second and most important limitation is the lack of validation of the developed score in an independent external cohort. Although internal validation using 1000 bootstrap resamples demonstrated low optimism, stable C-index values, and near-ideal calibration performance, prospective external validation studies are required to confirm the generalizability of the model across different populations and institutions. Accordingly, although the proposed nomogram provides a practical tool for individualized risk estimation, its routine clinical use should be considered preliminary until its performance is prospectively evaluated in independent clinical settings. Furthermore, because model development and reclassification analyses (NRI and IDI) were performed within the same cohort, the observed improvements in reclassification performance should be interpreted cautiously, as they may be overly optimistic until confirmed through independent external validation. From a methodological perspective, although the prognostic model was developed using a conventional regression-based variable selection strategy, we additionally evaluated a β-coefficient-weighted alternative scoring model, which demonstrated discriminative performance comparable to that of the simplified equal-weighted score. Given the comparable predictive performance of the two approaches and the greater clinical applicability of the equal-weighted score, the simplified scoring system was retained. Nevertheless, future studies using larger multicenter cohorts may benefit from penalized regression approaches, such as LASSO, to further optimize variable selection, reduce model overfitting, and enhance the robustness and generalizability of the model. In addition, despite the well-established prognostic significance of BRAF V600E mutation and MSI-H/dMMR status in metastatic colorectal cancer, the very small numbers of patients with these molecular alterations in our cohort prevented their reliable evaluation within the prognostic model. Evaluation in larger molecularly defined cohorts is warranted to determine whether integrating these biomarkers further improves prognostic performance. Finally, due to the retrospective nature of the study, the possibility that subclinical infections, concomitant inflammatory conditions, or undocumented medication use may have influenced inflammatory biomarkers cannot be entirely excluded.

## 5. Conclusions

This study developed and internally validated a practical and robust prognostic score for patients with mCRC integrating systemic inflammation, tumor dissemination, and clinical performance parameters. The developed model successfully stratified patients into distinct risk groups with marked survival differences and demonstrated superior discriminative and reclassification performance compared with both GPS and GRIm. In particular, by extending beyond conventional systems based solely on laboratory parameters and incorporating clinical and anatomical variables, the novel score may more comprehensively reflect the biological heterogeneity of mCRC. Therefore, the proposed score may represent a promising tool for risk stratification in patients with mCRC. Prospective multicenter external validation studies, particularly in patients receiving contemporary targeted therapies and immunotherapy, are warranted to confirm its generalizability and clinical utility before routine clinical implementation.

## Figures and Tables

**Table 1:** Baseline demographic, clinicopathological, and laboratory characteristics according to GPS and GRIm groups.

Variable	Overall (N = 310)	Low GRIm (0–1) (N = 262)	High GRIm (2–3) (N = 48)	*p* Value	GPS 0 (N = 137)	GPS 1 (N = 126)	GPS 2 (N = 47)	*p* Value
Age, years	61.0 (53.0–69.0)	61.0 (53.0–68.0)	63.5 (53.0–70.0)	0.512 †	62.0 (53.0–69.0)	59.0 (51.2–67.0)	65.0 (57.0–71.0)	0.042 †
**Sex, n (%)**								
Female	128 (41.3%)	111 (42.4%)	17 (35.4%)	0.427 ¶	61 (44.5%)	48 (38.1%)	19 (40.4%)	0.566 §
Male	182 (58.7%)	151 (57.6%)	31 (64.6%)		76 (55.5%)	78 (61.9%)	28 (59.6%)	
**ECOG PS, n (%)**								
ECOG 0–1	298 (96.1%)	254 (96.9%)	44 (91.7%)	0.097 ¶	134 (97.8%)	122 (96.8%)	42 (89.4%)	0.028 ¶
ECOG ≥ 2	12 (3.9%)	8 (3.1%)	4 (8.3%)		3 (2.2%)	4 (3.2%)	5 (10.6%)	
**BMI, kg/m^2^**	25.2 (22.8–29.1)	25.3 (23.0–29.4)	23.7 (21.3–27.0)	0.019 †	25.4 (22.8–29.4)	25.5 (22.9–29.7)	23.9 (21.1–25.8)	0.010 †
**Comorbidity, n (%)**								
No	161 (51.9%)	139 (53.1%)	22 (45.8%)	0.432 ¶	69 (50.4%)	71 (56.3%)	21 (44.7%)	0.348 §
Yes	149 (48.1%)	123 (46.9%)	26 (54.2%)		68 (49.6%)	55 (43.7%)	26 (55.3%)	
**Tumor location, n (%)**								
Right colon	72 (23.2%)	61 (23.3%)	11 (22.9%)	1.000 ¶	30 (21.9%)	29 (23.0%)	13 (27.7%)	0.478 ¶
Left colon	236 (76.1%)	199 (76.0%)	37 (77.1%)		107 (78.1%)	95 (75.4%)	34 (72.3%)	
Right + Left	2 (0.6%)	2 (0.8%)	0 (0.0%)		0 (0.0%)	2 (1.6%)	0 (0.0%)	
**Metastasis type, n (%)**								
Synchronous	245 (79.0%)	202 (77.1%)	43 (89.6%)	0.055 ¶	94 (68.6%)	108 (85.7%)	43 (91.5%)	<0.001 §
Metachronous	65 (21.0%)	60 (22.9%)	5 (10.4%)		43 (31.4%)	18 (14.3%)	4 (8.5%)	
**Number of metastatic sites, n (%)**								
1 site	167 (53.9%)	149 (56.9%)	18 (37.5%)	0.018 ¶	88 (64.2%)	59 (46.8%)	20 (42.6%)	0.004 §
≥2 sites	143 (46.1%)	113 (43.1%)	30 (62.5%)		49 (35.8%)	67 (53.2%)	27 (57.4%)	
**Liver metastasis, n (%)**								
No	89 (28.7%)	78 (29.8%)	11 (22.9%)	0.388 ¶	43 (31.4%)	34 (27.0%)	12 (25.5%)	0.639 §
Yes	221 (71.3%)	184 (70.2%)	37 (77.1%)		94 (68.6%)	92 (73.0%)	35 (74.5%)	
**Lung metastasis, n (%)**								
No	213 (68.7%)	183 (69.8%)	30 (62.5%)	0.315 ¶	101 (73.7%)	82 (65.1%)	30 (63.8%)	0.235 §
Yes	97 (31.3%)	79 (30.2%)	18 (37.5%)		36 (26.3%)	44 (34.9%)	17 (36.2%)	
**Peritoneal metastasis, n (%)**								
No	241 (77.7%)	206 (78.6%)	35 (72.9%)	0.450 ¶	107 (78.1%)	98 (77.8%)	36 (76.6%)	0.977 §
Yes	69 (22.3%)	56 (21.4%)	13 (27.1%)		30 (21.9%)	28 (22.2%)	11 (23.4%)	
**RAS status, n (%)**								
Wild-type	151 (48.7%)	132 (50.4%)	19 (39.6%)	0.396 ¶	71 (51.8%)	62 (49.2%)	18 (38.3%)	0.783 §
Mutant	129 (41.6%)	108 (41.2%)	21 (43.8%)		58 (42.3%)	52 (41.3%)	19 (40.4%)	
**Primary tumor surgery, n (%)**								
No	153 (49.4%)	130 (49.6%)	23 (47.9%)	0.876 ¶	50 (36.5%)	78 (61.9%)	25 (53.2%)	<0.001 §
Yes	157 (50.6%)	132 (50.4%)	25 (52.1%)		87 (63.5%)	48 (38.1%)	22 (46.8%)	
**Adjuvant chemotherapy, n (%)**								
No	235 (75.8%)	193 (73.7%)	42 (87.5%)	0.044 ¶	90 (65.7%)	104 (82.5%)	41 (87.2%)	<0.001 §
Yes	75 (24.2%)	69 (26.3%)	6 (12.5%)		47 (34.3%)	22 (17.5%)	6 (12.8%)	
**1 L biological agent, n (%)**								
None	32 (10.3%)	27 (10.3%)	5 (10.4%)	0.531 ¶	11 (8.0%)	16 (12.7%)	5 (10.6%)	0.583 ¶
Anti-VEGF	193 (62.3%)	160 (61.1%)	33 (68.8%)		88 (64.2%)	73 (57.9%)	32 (68.1%)	
Anti-EGFR	85 (27.4%)	75 (28.6%)	10 (20.8%)		38 (27.7%)	37 (29.4%)	10 (21.3%)	
Neutrophil/Lymphocyte ratio	3.0 (2.0–4.4)	2.8 (1.9–3.8)	6.7 (4.5–8.7)	<0.001 †	2.3 (1.6–3.2)	3.4 (2.4–4.8)	5.2 (3.4–7.5)	<0.001 †
Albumin, g/dL	4.1 (3.6–4.4)	4.1 (3.8–4.4)	3.2 (3.1–3.4)	<0.001 †	4.3 (4.1–4.5)	4.0 (3.7–4.4)	3.2 (2.8–3.3)	<0.001 †
CRP, mg/L	13.0 (3.8–35.0)	9.2 (3.0–23.0)	56.4 (34.8–95.6)	<0.001 †	3.4 (2.0–5.5)	22.8 (15.0–47.8)	61.0 (40.2–97.0)	<0.001 †
LDH, U/L	214.5 (178.0–314.8)	207.5 (172.0–270.8)	324.5 (287.5–424.5)	<0.001 †	193.0 (168.0–232.0)	249.5 (191.2–346.8)	299.0 (212.0–401.5)	<0.001 †
CEA, ng/mL	23.0 (4.5–127.0)	19.0 (4.2–111.5)	48.0 (16.0–272.0)	0.022 †	10.0 (3.1–60.0)	45.2 (7.5–356.8)	25.5 (5.5–115.2)	<0.001 †

Test symbols next to *p* values: † Mann–Whitney U test; § Pearson’s chi-square test; ¶ Fisher’s exact test (used for 2 × 2 tables and when expected cell count < 5). Continuous variables are presented as median (interquartile range [IQR]); categorical variables as n (%). Two-sided *p* < 0.05 considered statistically significant; *p* values < 0.001 reported as ‘<0.001’.

**Table 2:** Kaplan–Meier survival estimates for overall survival and progression-free survival according to GPS and GRIm groups.

**A. Overall Survival by GRIm Score Group (Kaplan–Meier Estimates)**						
**Group**	**N**	**Events**	**Median (95% CI), Months**	**1-Year Rate (95% CI), %**	**2-Year Rate (95% CI), %**	**3-Year Rate (95% CI), %**
Low GRIm (0–1)	262	201	23.7 (21.7–27.6)	79.7 (74.2–84.1)	49.5 (43.0–55.6)	30.6 (24.5–36.9)
High GRIm (2–3)	48	43	12.5 (9.6–18.6)	52.1 (37.2–65.0)	27.0 (15.1–40.5)	19.1 (8.9–32.2)
Log-rank *p*			<0.001 ‖			
**B. Progression-Free Survival by GRIm Score Group (Kaplan–Meier Estimates)**						
**Group**	**N**	**Events**	**Median (95% CI), Months**	**1-Year Rate (95% CI), %**	**2-Year Rate (95% CI), %**	**3-Year Rate (95% CI), %**
Low GRIm (0–1)	262	232	11.7 (10.5–13.1)	48.4 (42.1–54.4)	16.3 (11.9–21.2)	10.6 (6.9–15.2)
High GRIm (2–3)	48	46	9.2 (7.6–10.5)	33.3 (20.6–46.6)	11.6 (4.4–22.6)	4.6 (0.9–13.7)
Log-rank *p*			0.1080 ‖			
**C. Overall Survival by GPS Group (Kaplan–Meier Estimates)**						
**Group**	**N**	**Events**	**Median (95% CI), Months**	**1-Year Rate (95% CI), %**	**2-Year Rate (95% CI), %**	**3-Year Rate (95% CI), %**
**GPS 0**	137	99	29.7 (26.5–35.5)	90.3 (83.8–94.2)	65.2 (56.2–72.9)	39.4 (30.2–48.4)
**GPS 1**	126	100	20.4 (17.5–22.6)	71.6 (62.7–78.7)	35.7 (26.9–44.6)	23.4 (15.6–32.1)
**GPS 2**	47	45	10.7 (8.6–13.0)	42.6 (28.4–56.0)	17.0 (8.0–28.9)	12.2 (4.7–23.4)
Log-rank *p*			<0.001 ‖			
**D. Progression-Free Survival by GPS Group (Kaplan–Meier Estimates)**						
**Group**	**N**	**Events**	**Median (95% CI), Months**	**1-Year Rate (95% CI), %**	**2-Year Rate (95% CI), %**	**3-Year Rate (95% CI), %**
**GPS 0**	137	119	13.8 (11.6–15.6)	56.5 (47.6–64.4)	22.9 (16.0–30.6)	16.1 (10.0–23.3)
**GPS 1**	126	114	10.2 (8.9–12.4)	42.4 (33.6–51.0)	9.9 (5.2–16.4)	2.9 (0.6–8.5)
**GPS 2**	47	45	8.6 (7.1–9.7)	25.5 (14.2–38.5)	8.5 (2.7–18.6)	5.7 (1.2–15.5)
Log-rank *p*			<0.001 ‖			

Test symbol: ‖ Log-rank test. Median survival time and survival rates derived from Kaplan–Meier estimates with 95% confidence intervals. Two-sided *p* < 0.05 considered statistically significant.

**Table 3:** Multivariable Cox regression analyses for overall survival and progression-free survival according to GPS- and GRIm-based models.

**A. Multivariable Cox Regression for OS—Model Including GRIm Score (n = 280, Events = 221; C-Index = 0.675)**		
**Variable**	**aHR (95% CI)**	***p*** **Value**
Age (per year)	1.00 (0.99–1.02)	0.496 #
Sex: Male vs. Female	1.31 (0.98–1.76)	0.069 #
ECOG performance status: ECOG ≥ 2 vs. ECOG 0–1	3.28 (1.73–6.22)	<0.001 #
Tumor location: Right colon vs. Left colon	1.47 (1.03–2.08)	0.032 #
Tumor location: Right + Left vs. Left colon	1.38 (0.19–10.16)	0.751 #
Metastasis type: Synchronous vs. Metachronous	1.11 (0.73–1.69)	0.636 #
Number of metastatic sites: ≥2 sites vs. 1 site	2.20 (1.60–3.02)	<0.001 #
Liver metastasis: Yes vs. No	0.78 (0.53–1.13)	0.184 #
Peritoneal metastasis: Yes vs. No	1.10 (0.77–1.57)	0.605 #
RAS status: Mutant vs. Wild-type	1.20 (0.91–1.58)	0.201 #
Primary tumor surgery: Yes vs. No	0.58 (0.41–0.81)	0.001 #
GRIm group: High GRIm (2–3) vs. Low GRIm (0–1)	1.79 (1.22–2.64)	0.003 #
**B. Multivariable Cox Regression for PFS—Model Including GRIm Score (n = 280, Events = 252; C-Index = 0.587)**		
**Variable**	**aHR (95% CI)**	***p*** **Value**
Age (per year)	1.00 (0.99–1.01)	0.714 #
Sex: Male vs. Female	1.13 (0.86–1.48)	0.372 #
ECOG performance status: ECOG ≥ 2 vs. ECOG 0–1	2.00 (1.09–3.68)	0.026 #
Tumor location: Right colon vs. Left colon	1.27 (0.90–1.77)	0.171 #
Tumor location: Right + Left vs. Left colon	1.18 (0.16–8.54)	0.869 #
Metastasis type: Synchronous vs. Metachronous	0.86 (0.58–1.27)	0.449 #
Number of metastatic sites: ≥2 sites vs. 1 site	1.42 (1.05–1.91)	0.021 #
Liver metastasis: Yes vs. No	0.77 (0.55–1.07)	0.119 #
Peritoneal metastasis: Yes vs. No	1.14 (0.81–1.62)	0.458 #
RAS status: Mutant vs. Wild-type	1.11 (0.86–1.43)	0.422 #
Primary tumor surgery: Yes vs. No	0.69 (0.50–0.95)	0.022 #
GRIm group: High GRIm (2–3) vs. Low GRIm (0–1)	1.21 (0.84–1.75)	0.308 #
**C. Multivariable Cox Regression for OS—Model Including GPS Score (n = 280, Events = 221; C-Index = 0.695)**		
**Variable**	**aHR (95% CI)**	***p*** **Value**
Age (per year)	1.00 (0.99–1.02)	0.601 #
Sex: Male vs. Female	1.43 (1.06–1.93)	0.019 #
ECOG performance status: ECOG ≥ 2 vs. ECOG 0–1	3.05 (1.61–5.77)	<0.001 #
Tumor location: Right colon vs. Left colon	1.41 (1.00–2.00)	0.051 #
Tumor location: Right + Left vs. Left colon	1.12 (0.15–8.27)	0.912 #
Metastasis type: Synchronous vs. Metachronous	0.97 (0.63–1.49)	0.889 #
Number of metastatic sites: ≥2 sites vs. 1 site	2.09 (1.52–2.88)	<0.001 #
Liver metastasis: Yes vs. No	0.84 (0.58–1.22)	0.364 #
Peritoneal metastasis: Yes vs. No	1.19 (0.83–1.70)	0.352 #
RAS status: Mutant vs. Wild-type	1.22 (0.92–1.61)	0.162 #
Primary tumor surgery: Yes vs. No	0.59 (0.42–0.83)	0.003 #
**GPS group: GPS 1 vs. GPS 0**	1.52 (1.12–2.06)	0.006 #
**GPS group: GPS 2 vs. GPS 0**	2.88 (1.86–4.45)	<0.001 #
**D. Multivariable Cox Regression for PFS—Model Including GPS Score (n = 280, Events = 252; C-Index = 0.606)**		
**Variable**	**aHR (95% CI)**	***p*** **Value**
Age (per year)	1.00 (0.99–1.01)	0.830 #
Sex: Male vs. Female	1.17 (0.89–1.54)	0.256 #
ECOG performance status: ECOG ≥ 2 vs. ECOG 0–1	1.89 (1.03–3.48)	0.041 #
Tumor location: Right colon vs. Left colon	1.24 (0.88–1.75)	0.211 #
Tumor location: Right + Left vs. Left colon	0.97 (0.13–7.08)	0.978 #
Metastasis type: Synchronous vs. Metachronous	0.79 (0.54–1.18)	0.250 #
Number of metastatic sites: ≥2 sites vs. 1 site	1.32 (0.97–1.78)	0.075 #
Liver metastasis: Yes vs. No	0.81 (0.58–1.14)	0.221 #
Peritoneal metastasis: Yes vs. No	1.17 (0.82–1.66)	0.380 #
RAS status: Mutant vs. Wild-type	1.10 (0.85–1.42)	0.471 #
Primary tumor surgery: Yes vs. No	0.72 (0.52–1.00)	0.048 #
**GPS group: GPS 1 vs. GPS 0**	1.41 (1.06–1.88)	0.020 #
**GPS group: GPS 2 vs. GPS 0**	1.62 (1.07–2.45)	0.024 #

Test symbol: # Wald test from Cox proportional hazards regression. aHR = adjusted hazard ratio; CI = confidence interval. Reference category indicated after ‘vs’. Two-sided *p* < 0.05 considered statistically significant.

**Table 4:** Subgroup analyses of the prognostic impact of GPS and GRIm on overall survival.

Subgroup	N	Events	HR (95% CI)	*p* Value
**GRIm high (2–3) vs. low (0–1)—OS**				
**By biological agent (1 L)**				
Anti-VEGF	193	154	1.45 (0.96–2.19)	0.076 #
Anti-EGFR	85	68	6.58 (3.12–13.90)	<0.001 #
None	32	22	1.26 (0.45–3.55)	0.661 #
**By RAS status**				
Wild-type	151	120	2.16 (1.27–3.69)	0.005 #
Mutant	129	101	1.63 (1.00–2.67)	0.052 #
**By primary tumor location**				
Right colon	72	59	1.27 (0.62–2.60)	0.513 #
Left colon	236	184	1.97 (1.35–2.87)	<0.001 #
Right + Left	2	1	—	—
**GPS 2 vs. GPS 0–1—OS**				
**By biological agent (1 L)**				
Anti-VEGF	193	154	1.97 (1.32–2.94)	<0.001 #
Anti-EGFR	85	68	6.58 (3.12–13.90)	<0.001 #
None	32	22	1.76 (0.63–4.93)	0.279 #
**By RAS status**				
Wild-type	151	120	2.58 (1.53–4.36)	<0.001 #
Mutant	129	101	2.06 (1.23–3.45)	0.006 #
**By primary tumor location**				
Right colon	72	59	1.80 (0.94–3.43)	0.074 #
Left colon	236	184	2.54 (1.73–3.73)	<0.001 #
Right + Left	2	1	—	—

Test symbol: # Wald test from Cox proportional hazards regression. HR = hazard ratio; CI = confidence interval. Reference category indicated after ‘vs’. Two-sided *p* < 0.05 considered statistically significant.

**Table 5:** Final multivariable Cox model and point allocation of the novel prognostic score.

Variable	aHR (95% CI)	*p* Value
≥2 metastatic sites	1.53 (1.17–2.01)	0.002 #
CRP > 10 mg/L	1.45 (1.09–1.92)	0.011 #
Albumin < 3.5 g/dL	1.91 (1.34–2.73)	<0.001 #
ECOG ≥ 2	2.41 (1.32–4.41)	0.004 #
CEA ≥ 5 ng/mL	1.49 (1.09–2.04)	0.014 #
No primary tumor surgery	1.45 (1.11–1.91)	0.007 #
Peritoneal metastasis	1.54 (1.12–2.12)	0.008 #

Test symbol: # Wald test from Cox proportional hazards regression. aHR = adjusted hazard ratio; CI = confidence interval. Final variables retained after backward elimination from 11 univariate-significant candidates (univariate *p* < 0.10). All retained variables *p* < 0.05 in the final model. C-index of full Cox model: 0.688 (apparent), 0.678 (bootstrap-corrected, 500 resamples).

**Table 6:** Survival outcomes and discriminative performance of the novel prognostic score.

**A. Survival Outcomes by New-Score Risk Group**						
**Group**	**N**	**Events**	**Median OS (Months)**	**12-mo OS Rate, %**	**24-mo OS Rate, %**	**36-mo OS Rate, %**
Low (0–1)	67	39	40.9	95.2	72.8	59.7
Intermediate (2–3)	155	124	25.4	82.3	52.8	26.4
High (≥4)	88	81	11.5	47.9	13.6	10.5
**B. Discrimination—AUC at 24-Month OS and Harrell’s C-Index with Bootstrap Optimism Correction**						
**Score**	**AUC, 24-mo OS (95% CI)**	**C-Index (Apparent)**	**Optimism**	**C-Index (Corrected)**		
New Score	0.768 (0.710–0.820) **	0.681	−0.000	0.681		
GPS	0.697 (0.634–0.753) **	0.635	—	—		
GRIm	0.620 (0.559–0.679) **	0.597	—	—		
Full Cox model (continuous predictors)	—	0.688	+0.010	0.678		
**C. Pairwise AUC Comparison (DeLong’s Test for Paired ROC Curves)**						
**Comparison**	**AUC_1_**	**AUC_2_**	**DeLong *p***			
New Score vs. GPS	0.768	0.697	0.005 ††			
New Score vs. GRIm	0.768	0.620	<0.001 ††			

Risk groups: Low (0–1), Intermediate (2–3), High (≥4). Median OS estimated by Kaplan–Meier; landmark survival rates are point estimates. Test symbol: ** bootstrap 95% CI (1000 resamples for AUC). Optimism estimated by 500 bootstrap resamples (Steyerberg method). Corrected C-index = apparent—optimism. Test symbol: †† DeLong’s test for paired ROC curves. Two-sided *p* < 0.05 considered statistically significant.

## Data Availability

The datasets used and/or analyzed during the current study are available from the corresponding author on reasonable request.

## Supplementary Materials

The following supporting information can be downloaded at: https://www.mdpi.com/article/10.3390/jcm15135074/s1. Figure S1: Progression-free survival according to the novel prognostic score risk groups; Figure S2: Nomogram for individualized prediction of 24- and 36-month overall survival; Table S1: Univariable discriminative performance of GPS and GRIm assessed using Harrell’s C-index; Table S2: Reclassification analyses using net reclassification improvement (NRI) and integrated discrimination improvement (IDI) comparing the novel prognostic score with GPS and GRIm; Table S3: Internal bootstrap validation and calibration metrics of the novel prognostic score; Table S4: Comparison of the Equal-Weighted and β-Coefficient–Weighted Versions of the Novel Prognostic Score.
