# Supplementary material for: Development and Internal Validation of a Novel Prognostic Score in Metastatic Colorectal Cancer: A Comparative Retrospective Cohort Study with the Glasgow Prognostic Score and Gustave Roussy Immune Score
Source: J Clin Med. 2026 Jun 29;15(13):5074. doi: 10.3390/jcm15135074 (PMC13362653; doi:10.3390/jcm15135074)
Supplement: Supplementary file 1 [file jcm-15-05074-s001.zip › SUPPLEMENTARY TABLES.pdf]

SUPPLEMENTARY TABLES

Supplementary Table S1. Univariable discriminative performance of GPS and GRIm assessed using Harrell's C-index

Score	Univariable C-index, OS	Univariable C-index, PFS
**GPS**	0.635	0.582
GRIm	0.597	0.560

Harrell's C-index reflects discrimination of each score used as the only predictor in a Cox model. Higher value indicates better discrimination (0.5 = chance, 1.0 = perfect discrimination).

Supplementary Table S2. Reclassification analyses using net reclassification improvement (NRI) and integrated discrimination improvement (IDI) comparing the novel prognostic score with GPS and GRIm

Comparison	NRI (95% CI)	IDI (95% CI)
New Score vs GPS	+0.331 (+0.098,+0.559)	+0.072 (+0.022,+0.126)
New Score vs GRIm	+0.416 (+0.183,+0.642)	+0.148 (+0.081,+0.220)

Continuous Net Reclassification Improvement (NRI) and Integrated Discrimination Improvement (IDI) computed for 24-month OS, with rank-normalized scores. Bootstrap 95% CIs (1000 resamples). Positive values favor the new score.

Supplementary Table S3. Internal bootstrap validation and calibration metrics of the novel prognostic score

Score level	N	Predicted 24-mo mortality	Observed 24-mo mortality (95% CI)
Score = 0	22	0.229	0.200 (0.080-0.449)
Score = 1	45	0.323	0.310 (0.188-0.483)
Score = 2	86	0.443	0.358 (0.264-0.473)
Score = 3	69	0.586	0.613 (0.496-0.731)

Score = 4	54	0.753	0.844 (0.728-0.930)
Score = 5	28	0.894	0.876 (0.717-0.968)
Score = 6	6	0.985	1.000 (1.000-1.000)

Bootstrap-based calibration analysis using 1000 resamples (Steyerberg method). Apparent calibration slope = 1.001, intercept = -0.027 (ideal: slope = 1.0, intercept = 0). Optimism-corrected slope = 0.909, intercept = -0.029. These values indicate excellent calibration with minimal optimism. Predicted probabilities derived from the final Cox model; observed mortality estimated by Kaplan–Meier within each score level (95% CI shown).

Supplementary Table S4. Comparison of the Equal-Weighted and β-Coefficient–Weighted Versions of the Novel Prognostic Score

**A. Point allocation for the two scoring schemes**				
**Risk factor**	**β coefficient**	**Score A points**	**Score B points**	**Notes**
ECOG ≥2	0.880	1	2	Strongest predictor
Albumin <3.5 g/dL	0.645	1	2	Inflammation–nutrition axis
Peritoneal metastasis	0.434	1	1	
≥2 metastatic sites	0.428	1	1	
CEA ≥5 ng/mL	0.400	1	1	
No primary tumor surgery	0.370	1	1	
CRP >10 mg/L	0.370	1	1	
**Total score range**	—	**0–7**	**0–9**	**β/β_min, rounded**
**B. Discriminative performance comparison**				
**Performance metric**	**Score A (equal-weighted)**	**Score B (β-weighted)**	**Difference / p value**	
AUC for 24-month OS (95% CI)	0.768 (0.710–0.820)	0.767 (0.709–0.819)	DeLong p = 0.804	
Harrell C-index (apparent)	0.681	0.685	+0.004	
Optimism (500 bootstrap resamples)	0.000	+0.010	—	
C-index (optimism-corrected)	0.681	0.675	+0.006	
NRI vs GPS (95% CI)	+0.331 (0.098,0.559)	+0.345 (0.110,0.572)	Similar	
NRI vs GRIm (95% CI)	+0.416 (0.183,0.642)	+0.428 (0.199,0.659)	Similar	
IDI vs GPS (95% CI)	+0.072 (0.022,0.126)	+0.069 (0.020,0.123)	Similar	
IDI vs GRIm (95% CI)	+0.148 (0.081,0.220)	+0.151 (0.084,0.225)	Similar	

The two scoring schemes provide statistically indistinguishable discrimination (DeLong p = 0.804). The equal-weighted version (Score A) shows zero optimism in bootstrap internal validation, indicating that its data-independent weights eliminate overfitting in the weighting step. The β-weighted version (Score B) shows mild positive optimism (+0.010), reflecting expected shrinkage upon internal validation. Given the equivalent discrimination and superior protection against overfitting — together with greater bedside usability — the equal-weighted scheme (Score A) was selected as the primary score for clinical use. DeLong = DeLong's test for paired ROC curves; NRI = continuous Net Reclassification Improvement; IDI = Integrated Discrimination Improvement.
