# Supplementary figures and images for: Development and Internal Validation of a Novel Prognostic Score in Metastatic Colorectal Cancer: A Comparative Retrospective Cohort Study with the Glasgow Prognostic Score and Gustave Roussy Immune Score
Source: J Clin Med. 2026 Jun 29;15(13):5074. doi: 10.3390/jcm15135074 (PMC13362653; doi:10.3390/jcm15135074)

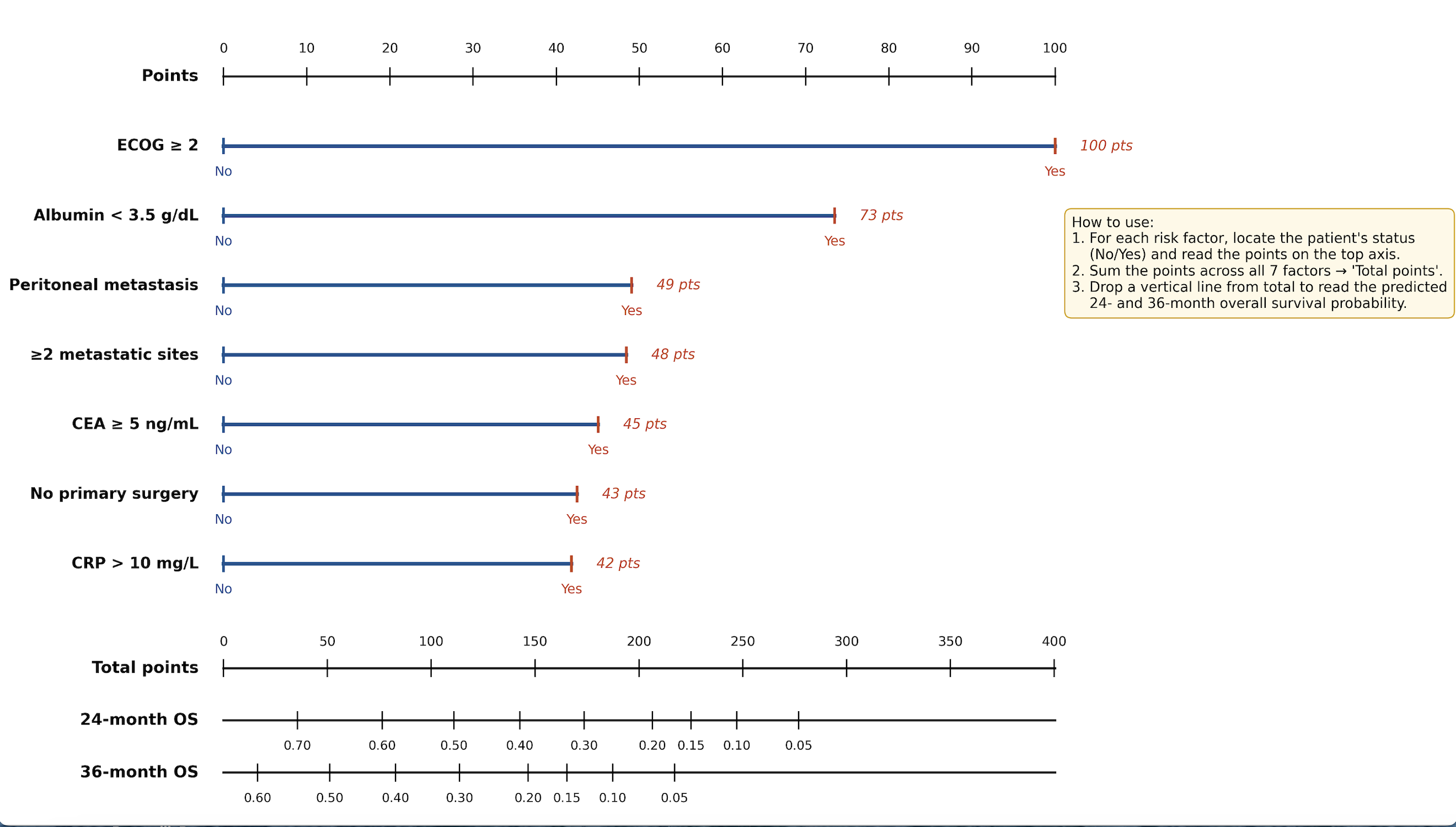

Supplement: Supplementary file 1 [file jcm-15-05074-s001.zip › Supplementary figure S2.png]

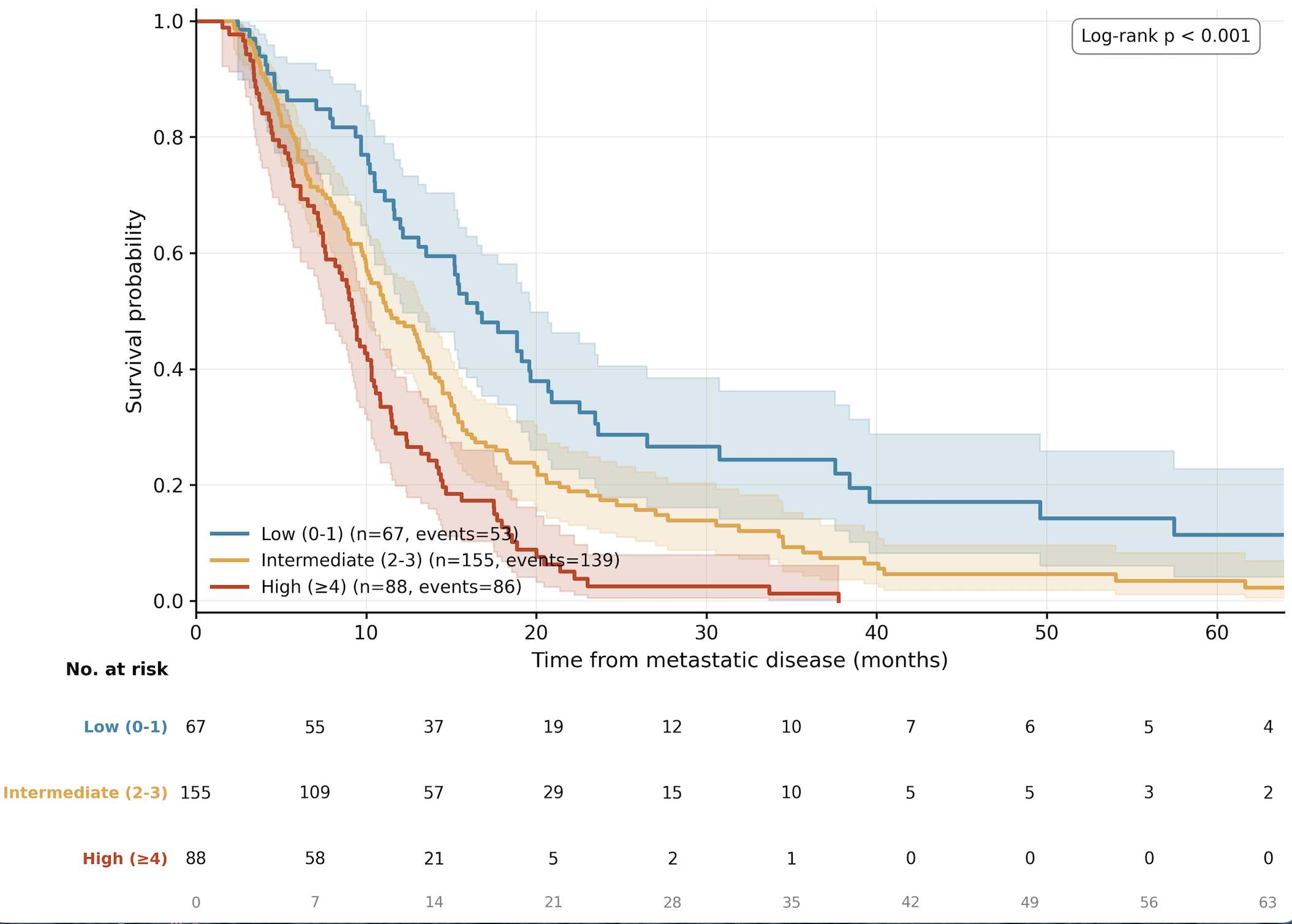

Supplement: Supplementary file 1 [file jcm-15-05074-s001.zip › Supplementary figure S1.png]
